# Exploratory data fusion of untargeted multimodal LC-HRMS with annotation by LCMS-TOF-ion mobility: White wine case study

**DOI:** 10.1177/14690667231164096

**Published:** 2023-03-21

**Authors:** Mpho Mafata, Maria Stander, Keabetswe Masike, Astrid Buica

**Affiliations:** 1 School for Data Science and Computational Thinking, 26697 Stellenbosch University, Stellenbosch, South Africa; 2 Department of Viticulture and Oenology, South African Grape and Wine Research Institute, 26697 Stellenbosch University, Stellenbosch, South Africa; 3 Central Analytical Facility, 26697 Stellenbosch University, Stellenbosch, South Africa

**Keywords:** Data fusion, multiple factor analysis, cluster analysis, agglomerative hierarchical clustering, exploratory multivariate analysis, high-resolution mass spectrometry, ion mobility, white wine

## Abstract

Applied sciences have increased focus on omics studies which merge data science with analytical tools. These studies often result in large amounts of data produced and the objective is to generate meaningful interpretations from them. This can sometimes mean combining and integrating different datasets through data fusion techniques. The most strategic course of action when dealing with products of unknown profile is to use exploratory approaches. For omics, this means using untargeted analytical methods and exploratory data analysis techniques. The current study aimed to perform data fusion on untargeted multimodal (negative and positive mode) liquid chromatography–high-resolution mass spectrometry data using multiple factor analysis. The data fusion results were interpreted using agglomerative hierarchical clustering on biplot projections. The study reduced the thousands of spectral signals processed to less than a hundred features (a primary parameter combination of retention time and mass-to-charge ratios, RT_m/z). The correlations between cluster members (samples and features from) were calculated and the top 10% highly correlated features were identified for each cluster. These features were then tentatively identified using secondary parameters (drift time, ion mobility constant and collision cross-section values) from the ion mobility spectra. These ion mobility (secondary) parameters can be used for future studies in wine chemical analysis and added to the growing list of annotated chemical signals in applied sciences.

## Introduction

In applied biological sciences, data is generated under many different categories such as quantitative *versus* qualitative, descriptive *versus* predictive, etc. Omics (e.g. metabolomics, proteomics, genomics, etc.) are a broad term covering data-centric studies in applied sciences which mainly focus on merging data science with analytical techniques.^1^ Of these analytical techniques, chemical analyses are most often used and can be categorised under targeted (specific analyte concentrations measured) and untargeted (wide range of spectral signals/chemical properties measured). When it comes to analyses of products of unknown profile space or for a more comprehensive investigation, untargeted analyses are often preferred.

There are many untargeted analyses for omics that are often hyphenated based on first separation (using phases such as gas, liquid, supercritical fluid, etc.) and then detection (e.g. ultraviolet-visible light (UV-Vis), mass spectrometry, fluorescence, etc.) methods. The choice of technique is dependent on the type of product analysed and its characteristics, and therefore its compatibility with different phases and detection methods. The choice is also dependent on the research problem under investigation. For example, investigations on the flavours of food products focus on volatile and/or non-volatile matrices depending on the research problem.^2^ In this regard, researchers can improve the analysis by either hyphenating the chromatography (e.g. GC/GC^3,4^) or the detection (*e.g*., UV-Vis-MS^4,5^).

High-resolution mass spectroscopy (HRMS) and tandem mass spectrometry (MS/MS) are two of the most widely used analytical techniques in metabolomics, with HRMS used often for untargeted analyses. Untargeted HRMS has been applied to studies in beverages for hypothesis testing/predictive studies on determining markers.^6^ Mass spectrometry-based omics^7^ are widely used and therefore have many databases and repositories/compendium such as the National Center for Biotechnology Information.^8^

Ion mobility spectrometry (IMS) can be coupled with HRMS, but it is seldom used because it is in its infancy, with most studies working on the method development^9–12^ and thus few focusing on its application to larger samples and/or using an untargeted approach.^13^ There are several limitations to working with IMS and therefore only a limited number of compounds are catalogued in the existing IMS databases. The method produces complex multidimensional data, from three-dimensional (m/z x drift time x ion abundance) in direct injection mode to four-dimensional data with the addition of chromatography (RT x m/z x drift time x ion abundance).^14^ Few software packages are able to read and analyse these complex datasets. Additionally, few software are capable of processing the data, given difficulties such as chromatographic shifts in retention time, a pivotal issue in the chemometrics of untargeted chromatographic data.^15^

In trying to address this limitation, studies can choose to focus on the chemical analyses or the data analyses. From the chemical analysis perspective, some studies have opted to reduce the dimensionality to just two sets of output through direct injection, thus excluding the chromatography.^13^ In these cases, the only separation is with the IMS whereby ions are separated in the gas phase based on their size, charge, and shape. Although this can be applied to model solutions and extracts, it is not always possible due to large discrepancies in concentrations and ionizability of compounds in complex matrices; therefore the chromatography is necessary.^16^ Alternatively, there are (a few) studies that reported on the use of ion mobility for the analysis of volatile and non-volatile compound in wine using targeted approaches.^10,17,18^ However, there have been no reports on untargeted approaches, whose potential has been noted by scholars.^19^ Given their potential, there is an opportunity to explore the non-volatile matrix composition by ion mobility. Furthermore, there is an opportunity to explore untargeted IMS as a stepping-stone to developing targeted approaches.

From the data analysis perspective, developers often create software packages to handle data specifically produced by certain chemical techniques (e.g. various forms (X) of chromatography mass spectrometry [XCMS],^20^ PARADISe for gas chromatography mass spectrometry [GCMS] data^21^) or for a specific manufacturer (*e.g*., MassLynx and Driftscope by Waters Corporation or MassHunter by Agilent technologies). This can be a limiting factor since these datasets may be of different formats and additional software may be needed to convert between data formats in the case of XCMS and mass spectrometry – data independent analysis (MS-DIAL).^22^ Developers then focus on software that can overcome these limitations by creating compilations of software (both licenced and opensource) to either convert between different data formats and/or simply read different types of data formats for more accessible omics.^23^

Since untargeted analyses generate large outputs of chemical signals and given the aforementioned issues when dealing with the data, the data science part of omics becomes necessary for contextual interpretations. From the large number of signals generated in untargeted analyses, finding suitable and appropriate data analysis techniques can lead to elucidating meaningful patterns from noise. These data analysis techniques can be broadly categorised into supervised (targeting certain outcomes, e.g., classification or prediction) and unsupervised (no imposed outcome) analyses. A good omics-based strategy should logically partner the chemical and data analyses accordingly.^24^ For example, an exploratory study of unknown product space should combine untargeted analyses with an unsupervised data strategy, and a predictive study of well-characterised products would use targeted analytical approaches with a supervised data strategy. These maintain an alignment of purpose and execution.

Omics, especially with exploratory strategies, are essential for breaking new ground on unknown spaces. Results of untargeted analyses can be tentatively annotated using existing libraries, compendia, and databases previously mentioned (such as National Institute of Standards and Technology [NIST], metabolite identification database [METLIN], Kyoto Encyclopedia of Genes and Genomes, etc.). Additionally, a compendium of ion mobility primary and secondary parameters was recently compiled from large number of published and/or peer-reviewed studies.^25^ This compendium encouraged the sharing and further development of ion mobility database(s). Explorative studies are hypothesis generating, meaning that they can elucidate multiple pathways to increase project success. Furthermore, databases showing the application of IMS in lipidomics,^26,27^ proteomics,^28^ mycotoxins,^29^ pesticides,^30,31^ and metabolomics^27,32^ studies have been noted.

Therefore, the aim of the current study was to conduct an exploratory data analysis of white wines using HRMS spectral data acquired in positive and negative modes, through multi-block data fusion and cluster analysis, followed by tentative identification using HRMS-IMS. The strategy compiled the three concepts for exploratory omics, namely, untargeted chemical analysis, unsupervised data analysis and tentative identification.

## Materials and methods

### Sampling and sample preparation

Twenty-seven samples (Table 1) were collected from various fast moving consumer goods liquor retail outlets in the Western Cape, South Africa. Three cultivars (Chenin blanc/CB, Chardonnay/CH, and Sauvignon blanc/SB) of either wooded or unwooded white wines were sourced. This generated five combinations of cultivar x winemaking (CB wooded/CBW and unwooded/CBU, Chardonnay wooded/CHW and unwooded/CHU, and SB unwooded/SBU only). It was ensured that cultivars and wine styles (wooded/unwooded) were acquired from the same producer/estate. The samples were centrifuged in 50 mL conical flasks at 3000 revolutions per minute/ 1160 relative centrifugal force in a Hermle Labortechnik (Z366) universal tabletop centrifuge (Lasec SA (Pty) Ltd, Cape Town, South Africa). The samples were then pipetted into 2 mL high-performance liquid chromatography (HPLC) vials, sparged with nitrogen gas, capped and stored in a refrigerator at approximately 4°C until analysis.

**Table 1.** Summary of sample identifiers.

Primary ID	Vintage	Wine of origin*	Cultivar	Wooded	Alcohol (%)	Additional information
CBU1	2018	Western Cape	Chenin blanc	No	13	Old vines, hand harvested, tank fermented
CBU2	2017	Western Cape	Chenin blanc	No	13.5	
CBU3	2018	Coastal region	Chenin blanc	No	13	Bush vines
CBU4	2018	Western Cape	Chenin blanc	No	12.5	Slow, controlled fermentation
CBU5	2018	Swartland	Chenin blanc	No	12.5	
CBU6	2018	Western Cape	Chenin blanc	No	13	
CBW1	2017	Stellenbosch	Chenin blanc	Yes	14	Old vine, hand harvested, barrel and tank fermented
CBW2	2017	Stellenbosch	Chenin blanc	Yes	13.5	Partial fermentation in oak barrels
CBW3	2017	Stellenbosch	Chenin blanc	Yes	13.5	Barrel fermented
CBW4	2017	Western Cape	Chenin blanc	Yes	13	
CBW5	2018	Western Cape	Chenin blanc	Yes	13	20% is aged on French oak barrels
CHU1	2018	Western Cape	Chardonnay	No	13	Hand harvested, tank fermented
CHU2	2018	Western Cape	Chardonnay	No	13	
CHU3	2018	Western Cape	Chardonnay	No	13.5	
CHU4	2018	Western Cape	Chardonnay	No	14	10 weeks thin lees
CHU5	2018	Western Cape	Chardonnay	No	13.5	
CHU6	2018	Western Cape	Chardonnay	No	13.5	
CHW1	2017	Western Cape	Chardonnay	Yes	13.5	Lightly oaked
CHW2	2017	Robertson	Chardonnay	Yes	14	Slightly wooded
CHW3	2017	Coastal region	Chardonnay	Yes	13	Matured in French oak barrels
CHW4	2018	Western Cape	Chardonnay	Yes	13.5	Well integrated nuances of fine French oak
CHW5	2017	Western Cape	Chardonnay	Yes	14	Lightly wooded
SBU1	2018	Robertson	Sauvignon blanc	No	12.5	
SBU2	2018	Western Cape	Sauvignon blanc	No	13	
SBU3	2018	Paarl	Sauvignon blanc	No	12.5	
SBU4	2018	Western Cape	Sauvignon blanc	No	12	Night harvested
SBU5	2018	Swartland	Sauvignon blanc	No	13	

*Wine of origin designation indicates that 100% of the grapes from which the wine is made comes from the designated area (WOSA.co.za).

### Instrumental analysis

**Figure 1.**
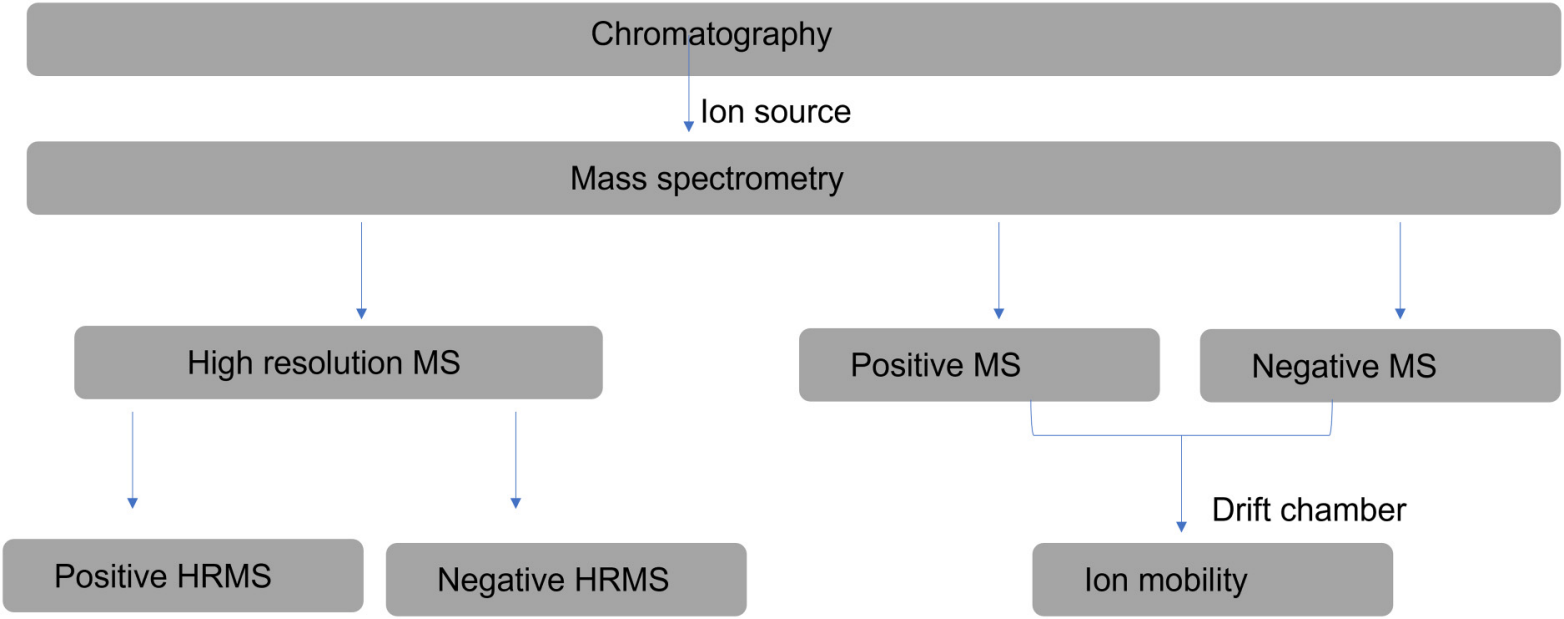
Instrumental workflow for the study experiment. MS, mass spectrometry.

The instrumental workflow (Figure 1) was designed such that the chromatography and MS conditions were the same for the HRMS and the ion mobility in order to have comparable results for metabolite annotation.

#### Liquid chromatography

For comparative consistency between the HRMS and IMS, the same liquid chromatography (LC) separation method was used. The conditions for separation, HRMS, and ion mobility were adapted from original methods by Masike et al.^33^ Chromatographic separations were done on a ultra-HPLC (Waters Corporation, Milford, MA, USA) equipped with a Synapt G2 quadrupole time-of-flight mass spectrometer (Q-TOF-MS, Waters Corporation, Milford, MA, USA), using an Acquity UPLC HSS T3 column (2.1 mm x 100 mm, 1.8 µm particle diameter, Waters Corporation, Milford, MA, USA) at a column temperature of 60°C. The run was 15 min using 0.1% formic acid (mobile phase A) and 0.1% formic acid in acetonitrile (mobile phase B). The gradient was 100% A for 30 s, then linearly increased to 22% B at 6 min, and then to 44% B over 6 min, and then over 50 s to 100% B for a 10 s wash step and returning to the initial conditions in 50 s; the column was re-equilibrated for 70 s at 100% A. The flow rate was 0.35 mL/min and the injection volume 2 µL.

#### High-resolution mass spectrometry

The settings of the Synapt system were as follows: a cone voltage of 20 V, desolvation temperature of 275°C, desolvation gas (nitrogen) was 650 L/h, and the capillary voltage was set at 3.5 kV. The instrument was calibrated with sodium formate and leucine enkephalin was used as lockspray reference compound for accurate mass determinations. HRMS data was generated using MS^E^ mode by scanning from *m/*z 120 to 1000 at a low collision energy (6 V) and a high collision energy scan at 30 V from *m/z* 40 to 1000 for fragmentation data. The MS acquisition was done with electrospray ionisation probe in positive (Pos) and negative (Neg) mode. Data processing was done using the MarkerLynx software (Waters Corporation, Milford, MA, USA) by peak detection with standard marker intensity threshold set at 200 counts and an additional set of negative mode below this threshold (LTNeg) to increase the number of markers. The data were integrated and extracted as a matrix of samples (observations) *versus* features (retention time_mass-to-charge ratio, RT_m/z) as the statistical variables, with values being in terms of ion abundance.

#### Ion mobility spectrometry

The travelling wave IMS was done in both positive and negative mode, using the same instrument, Synapt G2 Q-TOF-MS according to the method by Masike et al.^33^ A ion mobility (IMS) wave height of 30.2 V and a wave velocity of 387 m/s was used. The collision cross-section (CCS) values and ion mobility constant (K) were calculated using polyalanine calibrations. Data processing was done using Driftscope 2.9 software (Waters Corporation, Milford, MA, USA).

### Data handling

#### Data fusion and interpretation

Results were captured as three separate datasets: two according to the ionisation mode (Pos and Neg) at standard threshold and a third (LTNeg) captured below this threshold for the negative mode due to the relatively small number of peaks observed in the negative mode. The Pos and Neg datasets were integrated by multi-block data fusion using multiple factor analysis (MFA^34,35^). The MFA used Pearson correlation-based principal component analysis (PCA) on each dataset prior to data fusion. The MFA calculated is over N dimensions where N equals the number of observations (in this case the wine samples). Each data bock is subjected to weighing using the square root of its eigenvalue. The IMS data could not be integrated with the other datasets because it had an incompatible number of dimensions and could not be extracted using the MarkerLynx software. All statistical analyses were performed using XLSTAT software (Addinsoft, 2021, NY, USA. https://www.xlstat.com) and additional graphs were generated using Statistica13 (TIBCO, CA, USA).

#### Pattern recognition

The MFA scores and loadings patterns were analysed by agglomerative hierarchical clustering (AHC). The biplot matrix of samples and features was used for cluster analysis. The clustering was based on the Euclidean distance and done using single linkage. The cophenetic correlation coefficient was used to identify the optimal number of clusters. The clustering algorithm was imposed using a semisupervised approach, set to five classes corresponding with five subgrouping (i.e. cultivar x winemaking). For each cluster, the correlation coefficients of features to the class centroid were used as the delimiting parameter for selection of the optimal features. Selected for tentative annotation were the optimal features with correlation coefficients in the top 10%.

#### Tentative identification by IMS

Driftscope was used to visualise the IMS and select the important features as a square area of RT against m/z. The area was extracted as a separate MarkerLynx file retaining the drift time and used to identify the specific drift time related to the feature. Although manual and tedious given the number of features identified, this method assured relatively low chances of overlap in the drift time assigned. The polyalanine calibration (Supplemental Figure 1) was used to estimate the CCS values and ion mobility constant (K). The combination of m/z ratio and CCS values were compared with literature for tentative annotation.

## Results and discussion

### Data fusion

**Figure 2.**
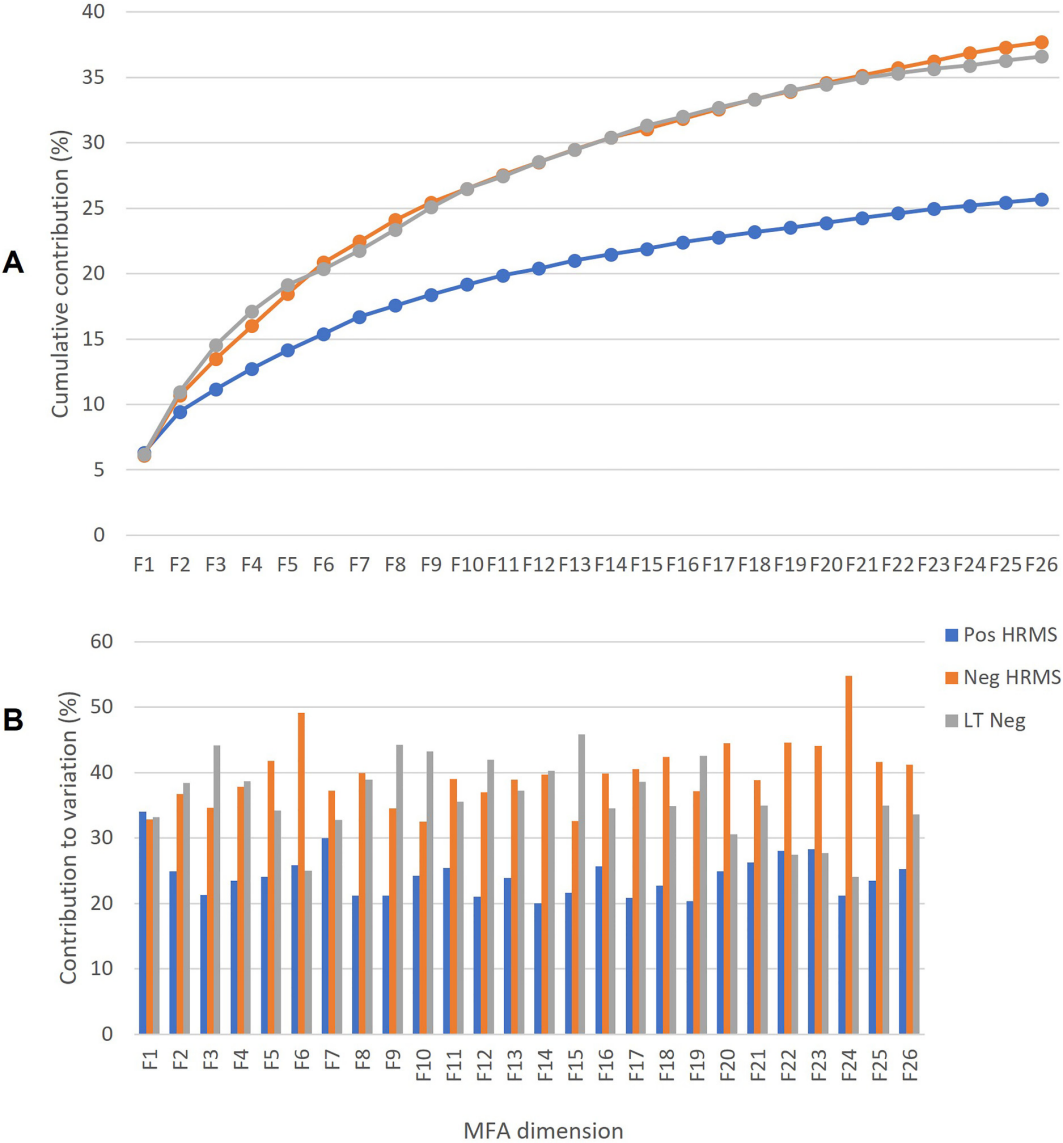
Percentage cumulative contribution to variation (A) for the MFA data fusion model per data block (Pos HRMS, Neg HRMS, and LTNeg) and contributions per dimension (B). HRMS, high-resolution mass spectrometry; LT, lower threshold; MFA, multiple factor analysis.

**Figure 3.**
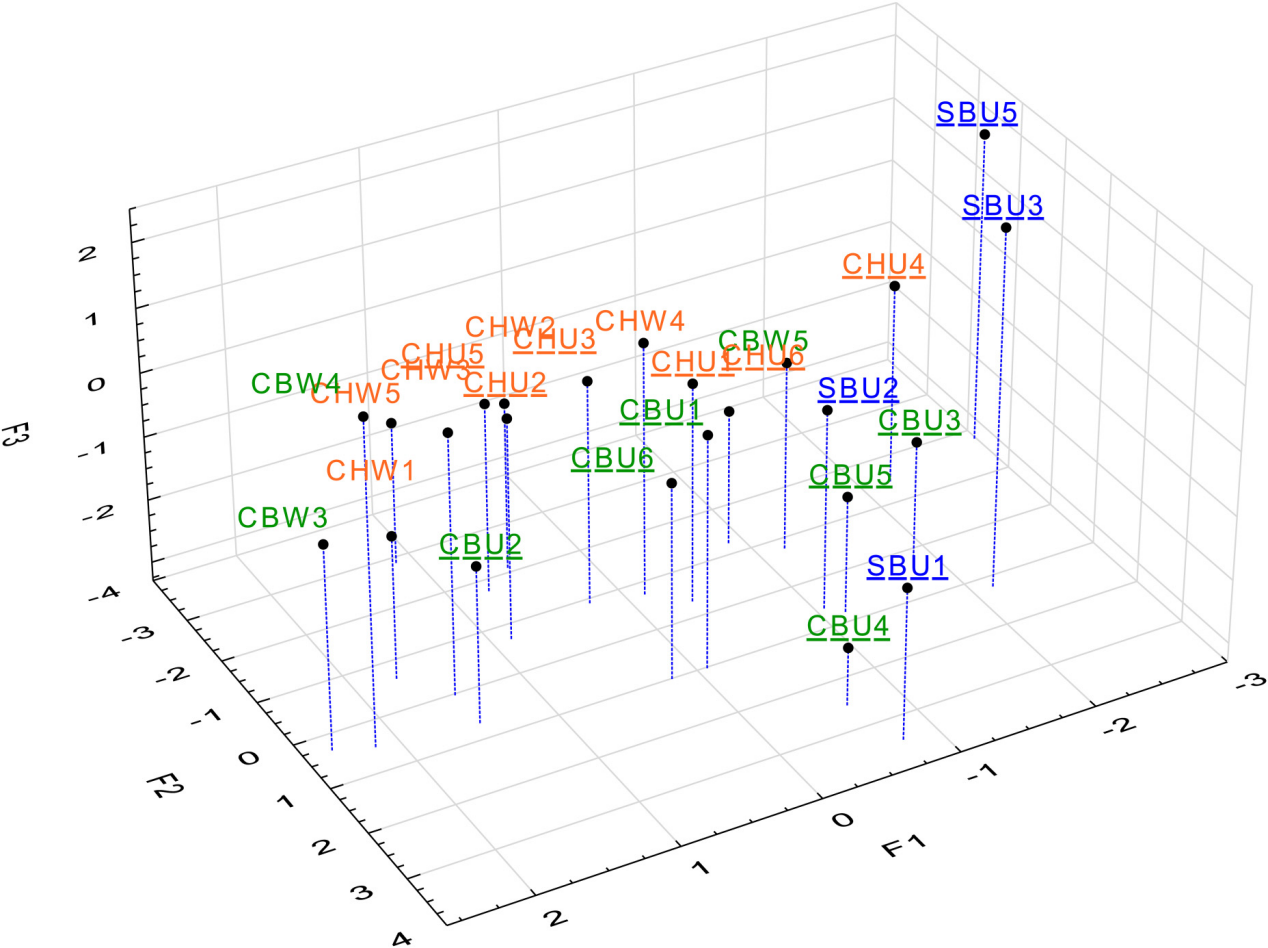
MFA scores plot for the first three dimensions (cumulative 39% EV) coloured according to cultivar. Cultivars are distinguished by colour and unwooded wines designated using underlined labels. EV, explained variance; MFA, multiple factor analysis.

The MFA model as well as the three data block models (Pos, Neg, and LTNeg HRMS) were evaluated using the performance indicators (Table 2 and Figure 2), followed by visual inspection of the MFA scores plot (Figure 3). The calculations of the model performance parameters were based on the distribution of the eigenvalue throughout each dimension.^36^ Efficient models will have a high rate of decrease in eigenvalue (rate of decay), meaning that the first few dimensions carry high proportions of the overall variation in the model. Therefore, the exponential decay function was calculated from the scree plot, and the performance parameters were derived from there.

**Table 2.** Summary of MFA data fusion model performance parameters.

	Exponential decay equation	R^2^	Total eigen value	Rate of decay (eigenvalue/ dimension)	Inflection point (dimension)	Inflection (%EV)	70%EV (dimension)	Eigenvalue ≥1 (dimension)	Eigenvalue 1 (%EV)	Number of variables
**Pos HRMS**	y = 94.142e^−0.114x^	0.7252	852	0.114	19	93.35	F8	All > 1	All > 1	853
**Neg HRMS**	y = 26.785e^−0.106x^	0.8708	240	0.106	21	94.98	F9	All > 2	All > 2	242
**LT Neg**	y = 13.527e^−0.126x^	0.9165	102	0.126	16	90.26	F9	F20	95.72	102
**MFA**	y = 1.5855e^−0.098x^	0.8127	15.3936	0.098	24	97.96	F10	F4	45.87	1197

EV, explained variance; HRMS, high-resolution mass spectroscopy; MFA, multiple factor analysis; LT, lower threshold.

The models of all three data blocks as well as the MFA were well fitted with R^2^ values above 0.7. The individual data blocks had PCA models with relatively higher efficiency compared to the MFA model, as indicated by a higher rate of decay (Table 2). This is an inherent feature of exploratory data fusion models^37^ and a consequence of the heterogeneity between different data blocks, common in applied sciences.^38^

Part of the assessment of the model performance is looking at the dimensions of optimal variability which uses three criteria for exploratory unsupervised analysis.^39^ The first is the inflection point which indicates a plateau in the rate of decay. The second is the first dimensions which cumulatively add up to 70% (or higher) explained variance (EV). The third is the cut-off of the dimensions with eigenvalues of one or less, since a dimension contributing less than one eigenvalue is considered less valuable than any given variable, and therefore ‘noise’.^39^ For the first criterion, the EVs in all models were higher than 90% at the inflection points. The third criterion reduced the dimensions only for the LTNeg PCA model and the MFA. This means that all dimensions in the Pos and Neg HRMS PCA models contained very little noise.^39^ The noise in the LTNeg model can be attributes to the instrumental noise when choosing lower threshold signals in the negative HRMS data. The inherent heterogeneity in MFA models as well as the LTNeg addition created the noise in the MFA model. Although it contains noise, it was not at a high enough level (only 6 out of 26 dimensions with eigenvalues below 1) to warrant concern since the data may still contain signals important to the study. The second criterion reduced the number of dimensions to a more manageable number compared to the inflection point, making it better for visual interpretation while retaining enough of the EV. Therefore, the 70% EV was chosen for further analysis (cluster analysis and visual summaries and interpretations).

In addition to the performance evaluation for the data fusion model and optimal dimension selection, the contribution of each data block was assessed (Figure 2, Supplemental Table 1). Each data block was weighted before data fusion so as not to skew the model, therefore the variability in the data fusion model coming from the three data blocks has been scaled. It is important to note that MFA, similar to multi-block PCA, is an orthogonal decomposition of the weighted data over all dimensions.^35,40^ Therefore, the contributions to variability coming from each data block will be different throughout each dimension (Figure 2B) and cumulatively across the entire model (Figure 2A).

The contributions to the variation in the overall model were higher for the negative mode HRMS (Neg HRMS: 25.7% EV and LTNeg: 36.6% EV) compared to the positive mode (Pos HRMS: 25.7% EV). For the first 10 dimensions, the positive mode (19% EV) again contributed the least to the cumulative variation of 72% EV compared to the equal contributions from the negative mode (26% EV from both Neg HRMS and LTNeg). This means that the positive mode had less variability than the negative mode. To find out why, the next step was to look at the patterns of the scores and the loadings (features) in the MFA.

Due to the high density of variables used in this study (1197 in total), visual inspection of the loading plots was more appropriately done using pattern recognition strategies, presented in the next section (Sample and feature correlations). Included here are only the evaluation of the scores plots visually (Figure 3) and statistically through pairwise regression vector (RV) coefficients (Supplemental Table 2) and cluster analysis (Figure 3). The third criterion (70% EV cut-off) was used to assess relevant patterns with little noise.

The RV coefficients between the MFA and each data block were high (MFA vs Pos HRMS: 0.95, vs Neg HRMS: 0.95, vs LTNeg: 0.96). This indicated that the MFA was representative of the sample configurations of each block. Although the model distributions for the Pos and Neg were different (Figure 2A), the RV coefficient values were high (Neg vs LTNeg – 0.85; Pos vs Neg – 0.86; and Pos vs LTNeg – 0.88). The Pos mode contained more signals than the Neg mode which were not all well correlated and therefore resulting in the relatively low model efficiency compared to the other models. The RV coefficients therefore may indicate that the cut-off criteria used was appropriate for reducing the noise in the Pos data. The visualised first three dimensions of the MFA scores plot showed variability in the distribution based both on cultivar and the winemaking (Figure 3). The most distinguishable pattern (visually) was the grouping of three SB samples (SBU3, SBU4 and SBU5). Given that these first three dimensions only captured 39% of the EV, and so as not incorporate visual bias in the interpretation, the cluster analysis was next applied.

**Figure 4.**
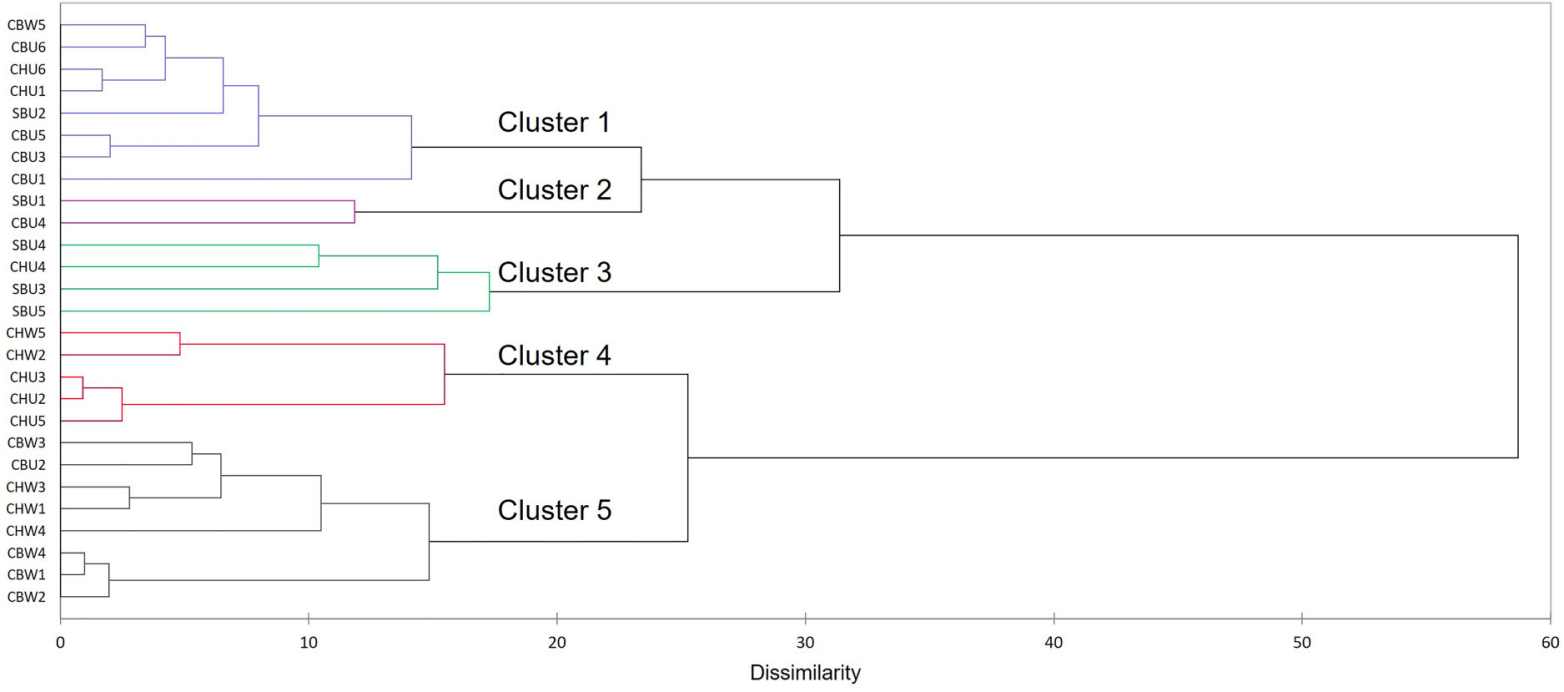
AHC dendrogram of the MFA scores plot for the optimal dimensions cumulatively adding up to 70% EV. AHC, agglomerative cluster analysis; MFA, multiple factor analysis.

The high RV coefficients between the MFA and individual PCA model scores (0.95–0.96) mean that the cluster analysis results were reliably representative of the sample configuration since the MFA conserved them. Given the limited amount of variation captured in the score plot (39% cumulative EV for the first three dimensions) and in order to derive meaningful patterns, the optimal dimensions (corresponding to 70% EV) were further used for cluster analysis. The AHC patterns will be discussed from top to bottom of the dendrogram (Figure 4). Clusters 1 and 2 contained a mixture of cultivars and winemaking styles (CBU, CBW, CHU, and SBU) with cluster 1 having predominantly CB samples. Cluster 3 had samples from the unwooded category, three SBU samples (out of five) and CHU4. The other two SBU samples clustered into two other separate groups (clusters 1 and 2). Cluster 4 was comprised of only CH samples but included both wooded and unwooded samples falling into separate subclusters. Cluster 5 was composed of only wooded CB and CH, with CHU2 as an exception (Figure 4). Within this cluster, was a subclass comprised only of CBW wines. Due to some of the clustering of samples according to either cultivar or winemaking previously discussed, the investigation continued to the exploration of the features correlated with these clusters in the next section.

### Sample and feature correlations

**Figure 5.**
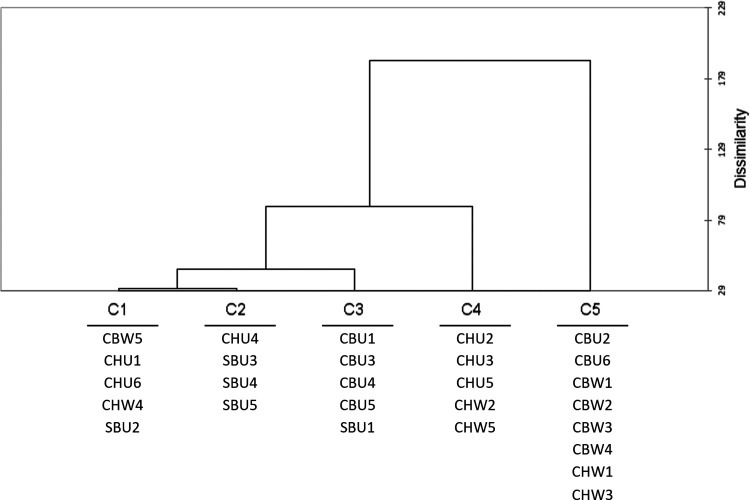
Clustering (AHC) dendrogram for the biplot map showing only the samples in each cluster. AHC, agglomerative cluster analysis.

In order to find the correlations between samples and features the biplot projection of scores and loadings from the MFA were submitted to cluster analysis (Figure 5). This concept is analogous to an unsupervised version of variable importance projections in supervised models such as partial least squares. The advantage of this strategy is that the loadings are projected onto the scores and clustered together to make it easier to find correlations given the large number of features. Additionally, biplot projections remove the visual bias for inferring correlations between scores and loadings in separate biplots. The same clustering conditions used for scores were used here: the first 10 dimensions as per optimal conditions (70% EV) and partitioning were again set at five clusters. The samples in each cluster will be discussed and compared to the scores clusters, followed by a discussion on the variable correlations in each cluster.

The dendrogram showed a cascade pattern of clustering, from most similar clusters (C1 and C2) to the most dissimilar (C3, C4 and C5) (Figure 5). The biplot projection clustering patterns (Figure 5) were similar to the scores clustering (Figure 4). C1 cluster contained a mixture of cultivars, wooded, and unwooded wines, similar to clusters 1 and 2 previously discussed for Figure 4. Due to the mixed categories (cultivar x winemaking) the feature correlations for this cluster were not carried forward. C2 contained the same members as cluster 3 previously discussed (SB and CHU4), and no features were contained in this cluster.

Clusters C3, C4 and C5 showed the more discernible patterns, therefore the feature correlations will be carried forward only on these three clusters. C3 contained unwooded CB samples with the exception of SBU1, which was not observed with the previous scores-only clustering (Figure 4). Hence, with the addition of the variables to the cluster analysis, these samples could be discriminated from the rest of the samples. C4 and C5 clustered the samples mainly according to cultivar with little majority of samples being of one winemaking style. That is, C4 contained only CH samples, three unwooded and two wooded, while C5 contained six CB samples of eight in total, but it could also be observed that six samples of eight were wooded. Overall, C3 showed mostly a cultivar x winemaking pattern (CB x U), C4 showed a cultivar pattern (all samples CH), and C5 showed an unclear cultivar and winemaking pattern (majority CB, majority W). These patterns will be incorporated into the following discussion on variable correlations.

**Figure 6.**
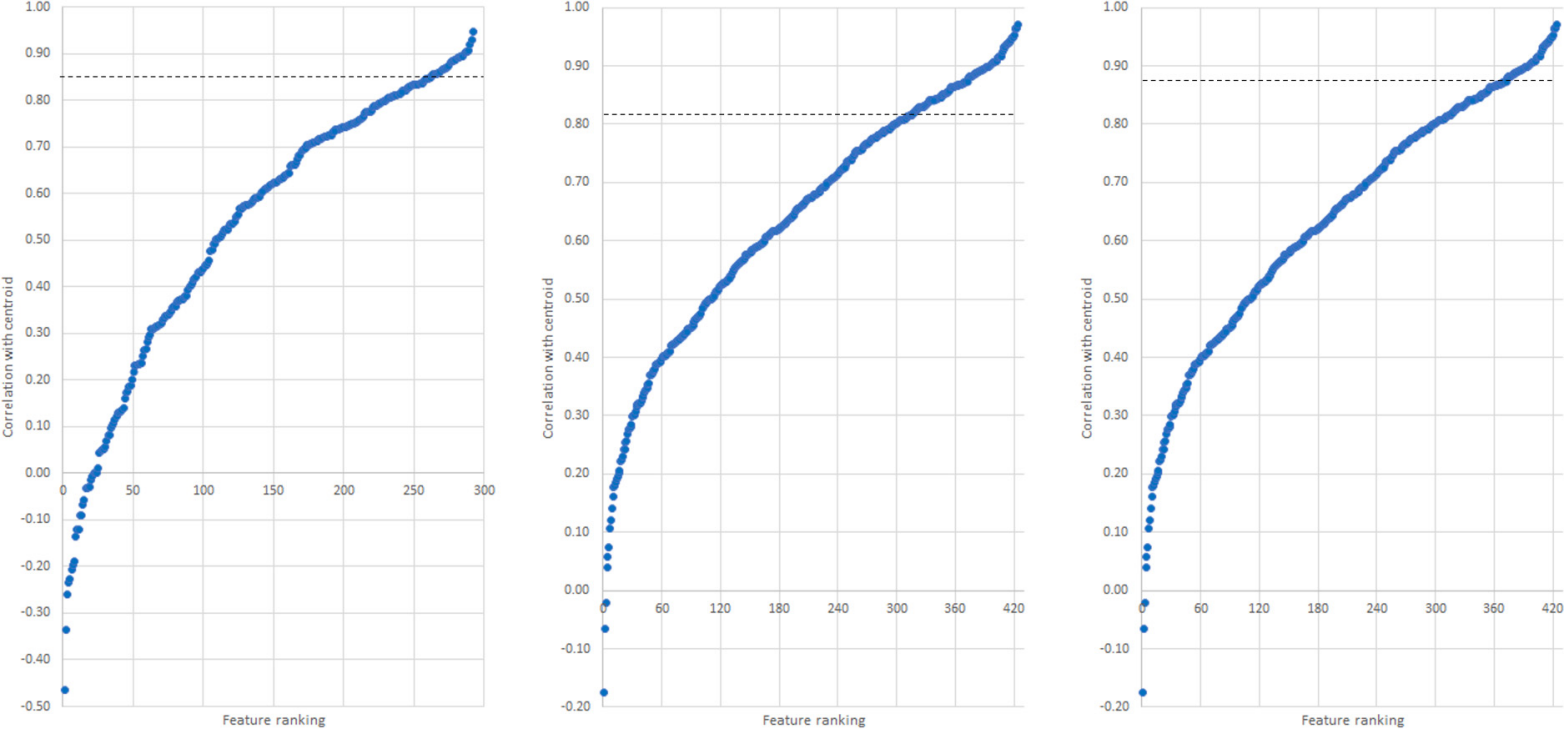
Feature correlations to centroid for (left to right), C3, C4, and C5. Dotted line indicates the cut-off point for the selected variables.

The correlations between features (samples and variables) and cluster centroid for clusters C3, C4 and C5 were explored through feature importance projections visualised using sigmoidal graphs (Figures 6). The projections rank the features from lowest to highest correlation coefficient (assigning a feature number). All samples were positively correlated with the cluster centroid; although the majority of variables were also positively correlated, some negatively correlated variables were observed for all three clusters. For finding the important variables, two selection criteria were considered: the first was highly correlated features (i.e. ≥ 0.9 correlation coefficient, signifying 10% highest correlation), and the second was the top number of variables with the highest correlation (top 10% of the total number of samples in each cluster). Although the second criterion resulted in more features selected, many had correlation coefficients below 0.60, therefore the first criterion was chosen.

Following implementation of the first criterion, the number of important variables identified was 7, 5 and 29 for C3, C4 and C5, respectively. The range of the correlation coefficients for these was from 0.90 to 0.95 for C3, to 0.92 for C4, and to 0.97 for C5. Given the low number of features identified, the optimal range criterion was adjusted for each cluster: instead of correlation coefficients from 0.90, it was adjusted to 0.85 for C3, 0.82 for C4 and 0.87 for C5. This adjustment was made based on the basis of the highest observed correlation coefficient in each cluster, therefore adjusting the 10% highest correlation coefficient based on the observed maxima of each cluster. The idea is to work from the observed maximum rather than the ideal/theoretical maximum. This resulted in the number of selected variables increasing from 7 to 30 for C3, from 5 to 21 for C4, and from 29 to 58 for C5. These features of importance (Figure 6) were all low molecular weight ions (m/z less than 600 Da), which corresponded with the known phenolic composition of white wines.^5^ These ions were then tentatively identified using their ion mobility signature.

### Tentative identification of important features

**Figure 7.**
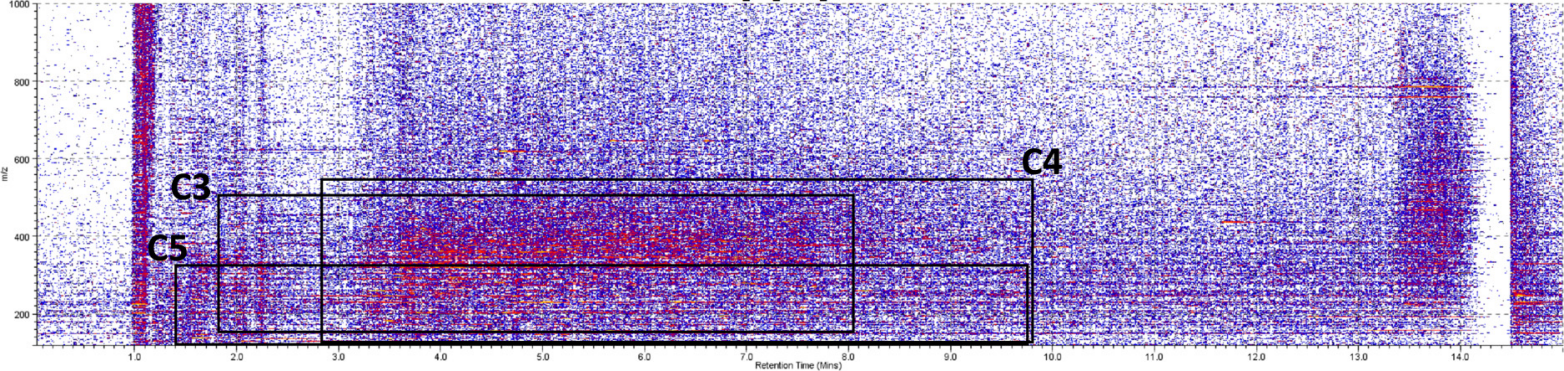
The two-dimensional (RT vs m/z) ion mobility spectra (CBU6). The squares indicate the ranges that the highly correlated features belonging to clusters C3, C4, and C5.

As an example of the typical ion mobility spectra of the wines, the two-dimensional ion mobility spectra of the wine (sample CBU6) showing RT versus drift time (t_d_) and drift time versus m/z are shown in Figure 7. From these spectra, the region of each feature identified was selected and in order to find the specific drift time of the related ions. Using polyalanine calibrations (Supplemental Table 3), the CCS values and ion mobility constant (K) were calculated and showed a reliable fit, with R^2^ of 0.9956. The values collected (m/z, t_d_, CCS, K, Supplemental Tables 4–6) were then used to tentatively identify the ions using the CCS compendium by Picache et al.^25^

Specifically, two of the calculated values were used for tentative identification based on literature values: the m/z ratio followed by CCS. The m/z ratio is more reliable since it is based on primary parameters (directly measured) while the CCS values are secondary parameters (calculated). CCS values were cross-checked against the literature methods compared to the method in the current study. Thus, the limitations of calculated CCS values were:

- The calibrant: CCS values can be calculated using different calibrants. The type of calibrant used is dependent on availability, the product types and the separation/detection method used. The calibrant must be compatible with the method conditions (e.g. normal vs reverse phase) and phase (e.g. gas, liquid, supercritical).
- The calibration error: how well the calibration was fitted and therefore can predict the CCS values of unknowns.
- Contextual plausibility: given that the product is wine, tentatively identified compounds that could not plausibly be present in wine were rejected.

**Figure 8.**
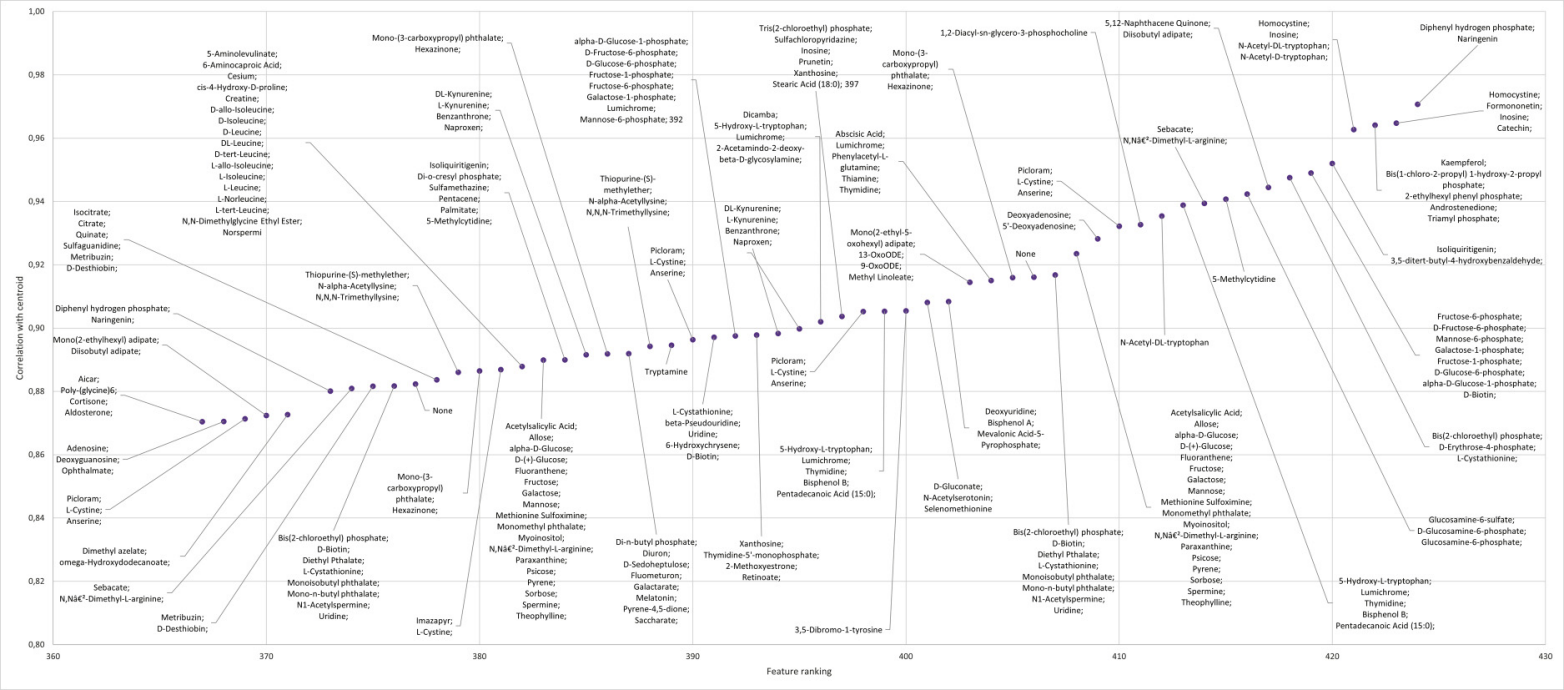
Annotation of the top features for cluster C5 ranked according to their correlation to centroids for all features in the cluster.

Using the above strategy, we attempted to annotate the features for each cluster. The ions are categorised under super classes, classes, and subclasses. For all three clusters, most of the ions belonged under two super classes, namely, organic acids and lipids. These are reasonable given that the chromatographic methods used in this study were set up for phenolic compounds. The annotated compounds are then plausible given the range of molecular mass of the ions identified and the separation method used in this study. Thus, by using the CCS values, the study was able to at least limit the category to which the selected ions belong. Annotation of features from cluster C3 (Supplemental Figure 3) and C4 (Supplemental Figure 4) had varying classes of compounds including fatty acids, phosphate esters, and amino acids. Annotation of features from cluster C5 (Figure 8) had many features compared to the other clusters, many of which were identified as carbohydrates (sugars), flavonoids and amino acids. Although the chromatography was directed toward phenolic compounds, there is plausibility of detecting amino acids, lipids, and carbohydrates since they do share similar chemical functional groups.^41^ For example, the hydroxycinnamates have similar functional groups to fatty acids and glycosylated anthocyanins have carbohydrate moieties.^41^ Additionally, phenolic compounds have been known to form covalent bonds to other proteins and carbohydrates, and may have similar ionisation patterns.^42^ Thus, it is plausible that they can be separated chromatographically in the same method. Additionally, these organic compounds are fermentation derived and have been measure in wines.^41,43,44^ Furthermore, although it is difficult to achieve from an instrumental perspective, this may indicate a need for direct injection of wine samples in order to be inclusive of other compound classes.

## Conclusion

The aim of this study was to conduct an exploratory data analysis of data generated from white wines using untargeted chemical data combined with unsupervised data analysis strategy. HRMS spectral data from positive and negative modes were integrated through MFA data fusion and cluster analysis applied using AHC on biplot projections. This was followed feature importance analysis on biplot projections on which tentative identification was done using HRMS-IMS. This strategy compiled the three concepts for exploratory omics: untargeted chemical analysis, unsupervised data analysis, tentative identification.

The MFA model performance parameters were different between Pos and Neg modes, however RV coefficients indicated high similarity in the sample configurations. The study used an optimisation strategy that imposed a cut-off of 70%EV for the first dimensions in the MFA to be used for cluster analysis. The cluster analysis showed some grouping of samples according to a mixture of cultivar and winemaking, with some clusters having categorical classifications by cultivar or by winemaking. To elucidate the features related to these clusters, clustering was then done on the biplot projections (samples and features) using the same optimisation strategy as with the scores only. Using the correlation coefficients with the cluster centroid, the features of importance were elucidated. These features were then annotated using their ion mobility spectra (primarily the m/z) and subsequent secondary calculations (CCS values), comparing them with published values. The study found multiple possible identities for many of the features belonging mostly to the organic compound categories: fatty acids, phosphate esters, carbohydrates and amino acids. The limit in these annotations is the published CCS values from phenolic compounds since many were from lipidomics and carbohydrate research. This reinforces the idea that more systematic effort should be put into expanding the IMS databases/knowledge. Although using the ion mobility is laborious, used in this nature and the annotation has its limitations, there are possibilities for hypothesis generation to narrow down the field of investigations, and therefore developing detailed analysis for classification of different product types.

## Supplemental Material

sj-docx-1-ems-10.1177_14690667231164096 - Supplemental material for Exploratory data fusion of untargeted multimodal LC-HRMS with annotation by LCMS-TOF-ion mobility: White wine case study Click here for additional data file. Supplemental material, sj-docx-1-ems-10.1177_14690667231164096 for Exploratory data fusion of untargeted multimodal LC-HRMS with annotation by LCMS-TOF-ion mobility: White wine case study by Mpho Mafata, Maria Stander, Keabetswe Masike and Astrid Buica in European Journal of Mass Spectrometry
