# Supplementary material for: Exploratory data fusion of untargeted multimodal LC-HRMS with annotation by LCMS-TOF-ion mobility: White wine case study
Source: Eur J Mass Spectrom (Chichester). 2023 Mar 21;29(2):111–22. doi: 10.1177/14690667231164096 (PMC10068406; doi:10.1177/14690667231164096)
Supplement: sj-docx-1-ems-10.1177_14690667231164096 - Supplemental material for Exploratory data fusion of untargeted multimodal LC-HRMS with annotation by LCMS-TOF-ion mobility: White wine case study [file sj-docx-1-ems-10.1177_14690667231164096.docx]

**Supplementary Files: Exploratory data analysis of the chemical fingerprint of white wine using untargeted LC-HRMS/MS and LCMS-TOF-Ion Mobility**

Mpho Mafata^1,2^*, Maria Aletta Stander^3^, Keabetswe Masike^3^, and Astrid Buica^1,2^*

^1^ School for Data Science and Computational Thinking, Stellenbosch University, South Africa

^2^ South African Grape and Wine Research Institute, Department of Viticulture and Oenology, Stellenbosch University, South Africa

^3^ Central Analytical Facility, Stellenbosch University, South Africa

^‡^ Current affiliation:

* Corresponding authors: [astrid.buica@gmail.com](mailto:astrid.buica@gmail.com) (A.B.) and [mafata@sun.ac.za](mailto:mafata@sun.ac.za) (M.M.)

# Supplementary figures


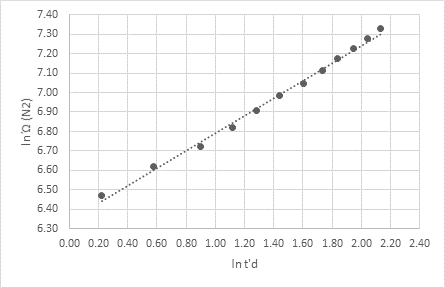


Supplementary Figure 1. Polyalanine calibration from calculation in Supplementary Table 3.


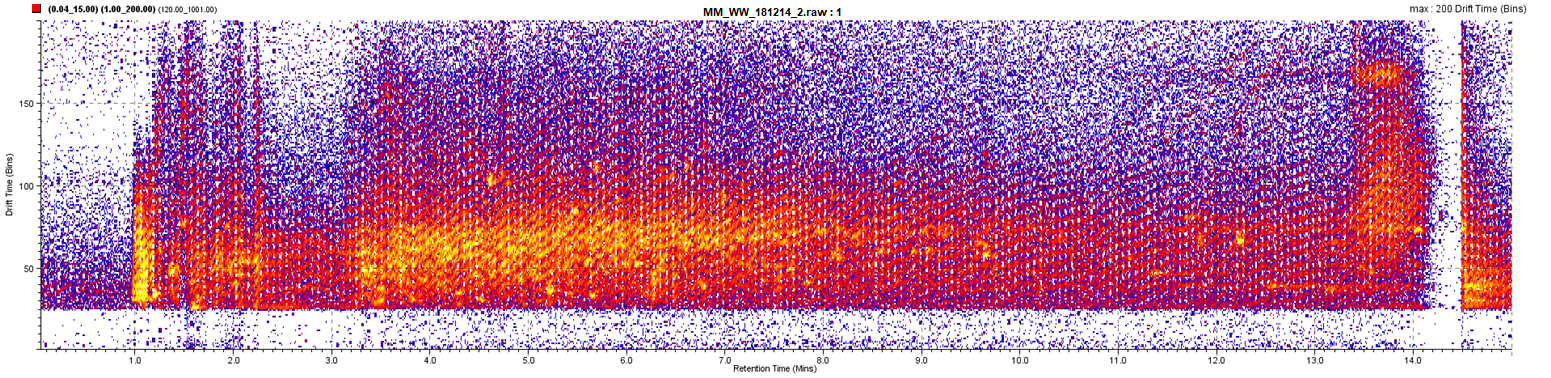


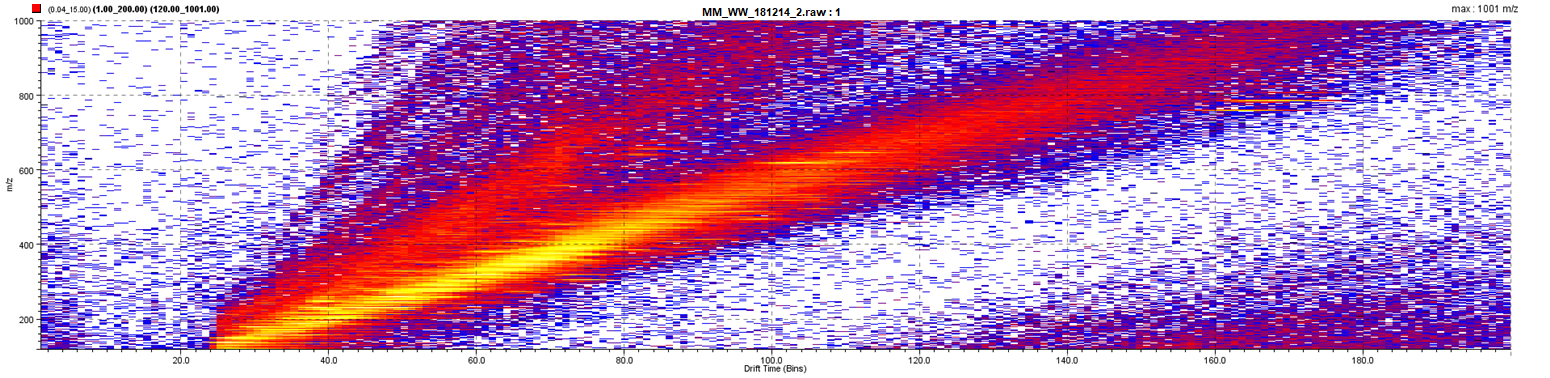


Supplementary Figure 2. Two-dimensional ion mobility spectra of white wine (sample CBU 6) showing RT vs Drift time (top) and drift time vs m/z (bottom).


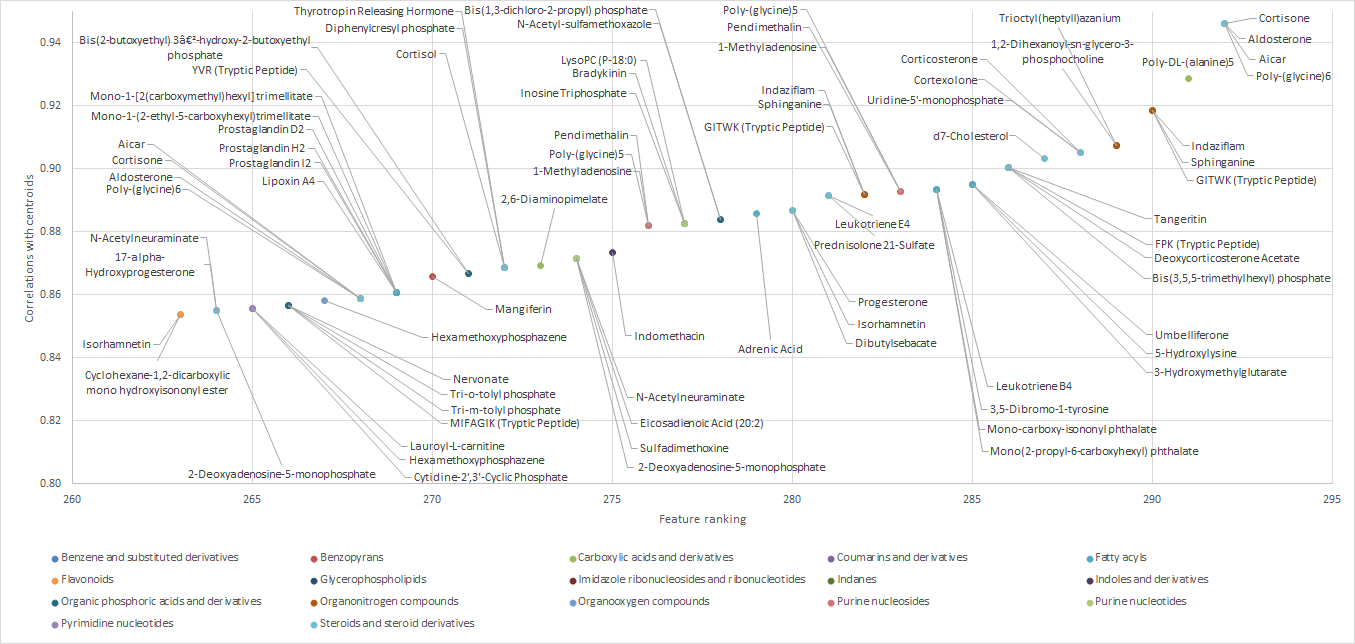


Supplementary Figure 3. Annotation of the top features for cluster C3 ranked according to their correlation to centroids for all features in the cluster.


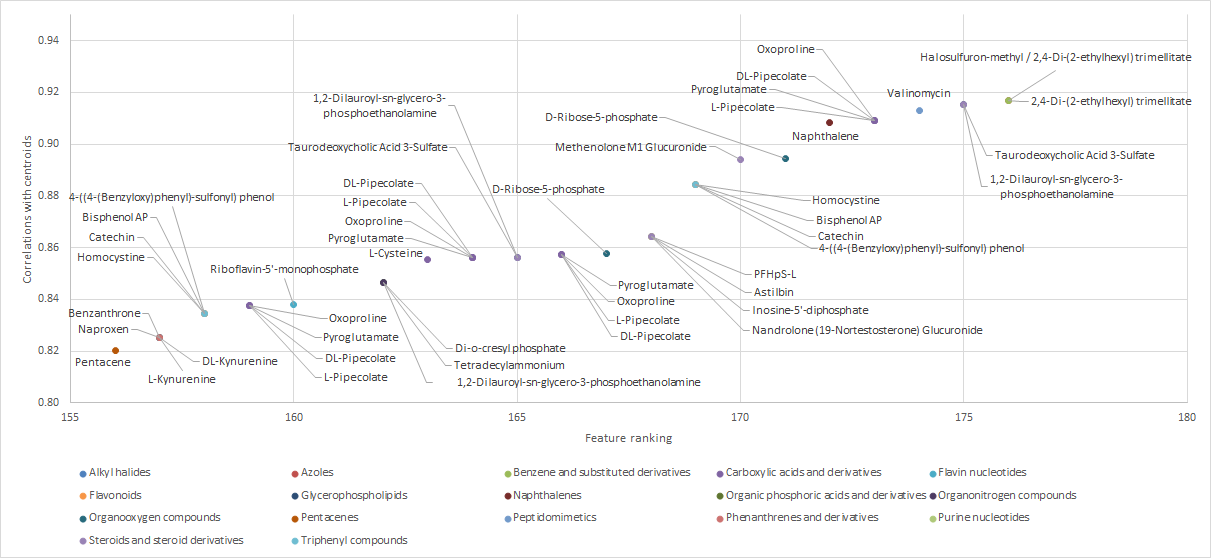


Supplementary Figure 4. Annotation of the top features for cluster C4 ranked according to their correlation to centroids for all features in the cluster.

# Supplementary tables

Supplementary Table 1. Component contribution to the variability in the MFA data fusion model.

| **Overall MFA model** | | **Data block** | **Cumulative contribution to MFA model over F1 to F10 (%)** | **Overall contribution to MFA model (%)** |
| --- | --- | --- | --- | --- |
| Total Eigenvalue | 15.39 | Pos HRMS | 19.17 | 25.69 |
| Cumulative Eigenvalue at F10 | 11.11 | Neg HRMS | 26.50 | 37.70 |
| Cumulative %EV at F10 | 72.17 | LT Neg | 26.49 | 36.61 |
|  |  | Summed variation | 72.17 | 100.00 |

Supplementary Table 2. Pairwise RV coefficients for MFA and individual data block model scores plots.

|  | **Pos HRMS** | **Neg HRMS** | **LT Neg** | **MFA** |
| --- | --- | --- | --- | --- |
| Pos HRMS | 1.0000 | 0.8610 | 0.8794 | 0.9519 |
| Neg HRMS | 0.8610 | 1.0000 | 0.8471 | 0.9505 |
| LT Neg | 0.8794 | 0.8471 | 1.0000 | 0.9562 |
| MFA | 0.9519 | 0.9505 | 0.9562 | 1.0000 |

Supplementary Table 3. Polyalanine calibration calculations.

| m/z | Driftime | t_D_ (ms) | t_transfer_ | t'd | ln t'd | µ (N_2_) | Ώ (N_2_) | ln Ώ (N_2_) | K (m/sec2) | CCS (Å) |
| --- | --- | --- | --- | --- | --- | --- | --- | --- | --- | --- |
| 161 | 1.61 | 1.6 | 1.3 | 1.2 | 0.22 | 23.86 | 645.73 | 6.47 | 304 | 132.20 |
| 232 | 2.14 | 2.1 | 1.8 | 1.8 | 0.57 | 24.99 | 749.86 | 6.62 | 214 | 150.00 |
| 303 | 2.82 | 2.8 | 2.5 | 2.5 | 0.90 | 25.64 | 832.42 | 6.72 | 155 | 164.40 |
| 374 | 3.42 | 3.4 | 3.1 | 3.1 | 1.12 | 26.06 | 917.28 | 6.82 | 125 | 179.70 |
| 445 | 3.98 | 4.0 | 3.6 | 3.6 | 1.28 | 26.35 | 1000.95 | 6.91 | 105 | 195.00 |
| 516 | 4.59 | 4.6 | 4.2 | 4.2 | 1.44 | 26.56 | 1079.79 | 6.98 | 90 | 209.50 |
| 587 | 5.35 | 5.4 | 5.0 | 5.0 | 1.60 | 26.73 | 1149.86 | 7.05 | 76 | 222.40 |
| 658 | 6.03 | 6.0 | 5.7 | 5.7 | 1.73 | 26.86 | 1228.88 | 7.11 | 67 | 237.10 |
| 729 | 6.66 | 6.7 | 6.3 | 6.3 | 1.84 | 26.97 | 1304.04 | 7.17 | 61 | 251.10 |
| 800 | 7.37 | 7.4 | 7.0 | 7.0 | 1.94 | 27.06 | 1377.45 | 7.23 | 54 | 264.80 |
| 871 | 8.10 | 8.1 | 7.8 | 7.7 | 2.04 | 27.13 | 1443.94 | 7.28 | 49 | 277.20 |
| 942 | 8.82 | 8.8 | 8.5 | 8.4 | 2.13 | 27.20 | 1520.74 | 7.33 | 45 | 291.60 |

Supplementary Table 4. Ion mobility drift calculations for cluster 3 (C3).

| **Primary ID** | **m/z** | **RT** | **t_D_ (ms)** | **t_transfer_** | **t'd** | **ln t'd** | **µ (N_2_)** | **ln Ώ (N_2_)** | **Ώ (N_2_)** | **CCS (Å)** | **Ion mobility K (m/sec2)** |
| --- | --- | --- | --- | --- | --- | --- | --- | --- | --- | --- | --- |
| Pos_49 | 347.23 | 1.83 | 3.46 | 3.12 | 3.09 | 1.13 | 25.92 | 6.85 | 945.19 | 185.67 | 123 |
| Pos_97 | 374.24 | 2.18 | 3.78 | 3.44 | 3.41 | 1.23 | 26.06 | 6.90 | 987.88 | 193.53 | 111 |
| Pos_247 | 375.22 | 3.98 | 3.73 | 3.39 | 3.36 | 1.21 | 26.06 | 6.89 | 981.33 | 192.23 | 113 |
| Pos_279 | 390.19 | 4.11 | 3.83 | 3.49 | 3.46 | 1.24 | 26.13 | 6.90 | 994.29 | 194.51 | 110 |
| Pos_288 | 304.19 | 4.16 | 3.94 | 3.60 | 3.57 | 1.27 | 25.64 | 6.92 | 1008.80 | 199.21 | 106 |
| Neg_89 | 302.17 | 4.19 | 3.19 | 2.85 | 2.82 | 1.04 | 25.63 | 6.81 | 907.38 | 179.23 | 135 |
| Pos_320 | 318.20 | 4.33 | 3.19 | 2.85 | 2.82 | 1.04 | 25.74 | 6.81 | 907.29 | 178.83 | 135 |
| Pos_404 | 213.16 | 4.80 | 2.16 | 1.82 | 1.80 | 0.59 | 24.75 | 6.61 | 740.57 | 148.85 | 211 |
| Pos_462 | 304.19 | 5.10 | 3.94 | 3.60 | 3.57 | 1.27 | 25.64 | 6.92 | 1008.80 | 199.21 | 106 |
| Pos_468 | 376.17 | 5.13 | 3.73 | 3.39 | 3.36 | 1.21 | 26.07 | 6.89 | 981.33 | 192.21 | 113 |
| Pos_608 | 302.21 | 6.05 | 3.19 | 2.85 | 2.82 | 1.04 | 25.63 | 6.81 | 907.38 | 179.23 | 135 |
| Pos_666 | 362.21 | 6.55 | 3.51 | 3.17 | 3.14 | 1.14 | 26.00 | 6.86 | 951.96 | 186.71 | 121 |
| LTNeg_81 | 330.24 | 6.64 | 3.46 | 3.12 | 3.09 | 1.13 | 25.82 | 6.85 | 945.28 | 186.04 | 123 |
| Pos_687 | 423.22 | 6.74 | 4.16 | 3.82 | 3.79 | 1.33 | 26.27 | 6.94 | 1035.73 | 202.09 | 100 |
| Pos_691 | 373.24 | 6.77 | 3.94 | 3.60 | 3.57 | 1.27 | 26.05 | 6.92 | 1008.46 | 197.58 | 106 |
| Pos_693 | 302.21 | 6.79 | 3.19 | 2.85 | 2.82 | 1.04 | 25.63 | 6.81 | 907.38 | 179.23 | 135 |
| Pos_695 | 316.22 | 6.82 | 3.40 | 3.06 | 3.03 | 1.11 | 25.73 | 6.84 | 937.06 | 184.74 | 125 |
| Pos_698 | 185.13 | 6.83 | 1.84 | 1.50 | 1.48 | 0.39 | 24.33 | 6.52 | 678.34 | 137.53 | 257 |
| LTNeg_87 | 332.22 | 6.85 | 3.40 | 3.06 | 3.03 | 1.11 | 25.83 | 6.84 | 936.98 | 184.36 | 125 |
| Pos_708 | 332.22 | 6.89 | 3.46 | 3.12 | 3.09 | 1.13 | 25.83 | 6.85 | 945.27 | 186.00 | 123 |
| Pos_709 | 360.21 | 6.89 | 3.73 | 3.39 | 3.36 | 1.21 | 25.99 | 6.89 | 981.40 | 192.52 | 113 |
| Pos_710 | 359.23 | 6.92 | 3.78 | 3.44 | 3.41 | 1.23 | 25.98 | 6.90 | 987.95 | 193.83 | 111 |
| Neg_200 | 314.21 | 7.10 | 3.24 | 2.90 | 2.87 | 1.06 | 25.71 | 6.82 | 914.51 | 180.34 | 132 |
| Pos_737 | 360.21 | 7.19 | 3.67 | 3.33 | 3.30 | 1.19 | 25.99 | 6.88 | 973.48 | 190.97 | 115 |
| Pos_738 | 530.32 | 7.19 | 5.18 | 4.84 | 4.81 | 1.57 | 26.60 | 7.05 | 1152.60 | 223.47 | 79 |
| Pos_741 | 453.24 | 7.24 | 4.32 | 3.98 | 3.95 | 1.37 | 26.38 | 6.96 | 1055.06 | 205.43 | 96 |
| LTNeg_93 | 437.24 | 7.39 | 4.16 | 3.82 | 3.79 | 1.33 | 26.32 | 6.94 | 1035.68 | 201.87 | 100 |
| Pos_755 | 437.24 | 7.42 | 4.32 | 3.98 | 3.95 | 1.37 | 26.32 | 6.96 | 1055.13 | 205.67 | 96 |
| Pos_758 | 344.26 | 7.54 | 3.67 | 3.33 | 3.30 | 1.19 | 25.90 | 6.88 | 973.56 | 191.30 | 115 |
| Pos_785 | 358.27 | 8.02 | 3.83 | 3.49 | 3.46 | 1.24 | 25.98 | 6.90 | 994.44 | 195.12 | 110 |

Supplementary Table 5. Ion mobility drift calculations for cluster 4 (C4).

| **Primary ID** | **Feature ID** | **m/z** | **RT** | **t_D_ (ms)** | **t_transfer_** | **t'd** | **ln t'd** | **µ (N_2_)** | **ln Ώ (N_2_)** | **Ώ (N_2_)** | **CCS (Å)** | **Ion mobility K (m/sec2)** |
| --- | --- | --- | --- | --- | --- | --- | --- | --- | --- | --- | --- | --- |
| Pos_24 | 1.44_231.1351 | 231.14 | 1.44 | 2.38 | 2.04 | 2.02 | 0.70 | 24.98 | 6.66 | 779.90 | 156.04 | 188 |
| Pos_47 | 1.83_278.1236 | 278.12 | 1.83 | 2.75 | 2.41 | 2.38 | 0.87 | 25.44 | 6.73 | 840.96 | 166.72 | 159 |
| Neg_43 | 2.75_128.0338 | 128.03 | 2.75 | 1.40 | 1.06 | 1.04 | 0.04 | 22.98 | 6.36 | 579.51 | 120.89 | 365 |
| Pos_116 | 2.75_130.0508 | 130.05 | 2.75 | 1.35 | 1.01 | 0.99 | -0.01 | 23.04 | 6.34 | 566.80 | 118.07 | 383 |
| Neg_48 | 3.13_128.0340 | 128.03 | 3.13 | 1.40 | 1.06 | 1.04 | 0.04 | 22.98 | 6.36 | 579.51 | 120.89 | 365 |
| Pos_154 | 3.48_130.0511 | 130.05 | 3.48 | 1.35 | 1.01 | 0.99 | -0.01 | 23.04 | 6.34 | 566.80 | 118.07 | 383 |
| Pos_204 | 3.77_144.1029 | 144.10 | 3.77 | 1.57 | 1.23 | 1.21 | 0.19 | 23.45 | 6.43 | 620.05 | 128.05 | 314 |
| Neg_138 | 5.37_577.1337 | 577.13 | 5.37 | 5.45 | 5.11 | 5.07 | 1.62 | 26.71 | 7.07 | 1181.14 | 228.54 | 75 |
| Neg_153 | 5.85_289.0711 | 289.07 | 5.85 | 3.02 | 2.68 | 2.65 | 0.98 | 25.53 | 6.78 | 882.47 | 174.64 | 143 |
| Neg_159 | 6.04_577.1340 | 577.13 | 6.04 | 5.45 | 5.11 | 5.07 | 1.62 | 26.71 | 7.07 | 1181.14 | 228.54 | 75 |
| Pos_610 | 6.05_579.1503 | 579.15 | 6.05 | 5.45 | 5.11 | 5.07 | 1.62 | 26.71 | 7.07 | 1181.13 | 228.52 | 75 |
| Neg_169 | 6.30_229.0970 | 229.10 | 6.30 | 2.32 | 1.98 | 1.96 | 0.67 | 24.96 | 6.65 | 769.39 | 154.02 | 194 |
| Neg_175 | 6.40_289.0708 | 289.07 | 6.40 | 3.02 | 2.68 | 2.65 | 0.98 | 25.53 | 6.78 | 882.47 | 174.64 | 143 |
| Neg_180 | 6.52_229.0968 | 229.10 | 6.52 | 2.32 | 1.98 | 1.96 | 0.67 | 24.96 | 6.65 | 769.39 | 154.02 | 194 |
| Neg_187 | 6.82_433.2065 | 433.21 | 6.82 | 4.21 | 3.87 | 3.84 | 1.35 | 26.31 | 6.95 | 1041.82 | 203.13 | 99 |
| Neg_208 | 7.40_449.2019 | 449.20 | 7.40 | 4.21 | 3.87 | 3.84 | 1.34 | 26.36 | 6.95 | 1041.75 | 202.90 | 99 |
| Neg_211 | 7.54_477.0671 | 477.07 | 7.54 | 4.27 | 3.93 | 3.90 | 1.36 | 26.45 | 6.96 | 1048.94 | 203.94 | 98 |
| Pos_760 | 7.56_479.0812 | 479.08 | 7.56 | 4.37 | 4.03 | 4.00 | 1.39 | 26.46 | 6.97 | 1060.95 | 206.26 | 95 |
| Pos_823 | 9.75_567.3349 | 567.33 | 9.75 | 5.08 | 4.74 | 4.70 | 1.55 | 26.69 | 7.04 | 1141.62 | 220.98 | 81 |
| Pos_825 | 9.76_129.0655 | 129.07 | 9.76 | 1.40 | 1.06 | 1.04 | 0.04 | 23.01 | 6.36 | 579.49 | 120.80 | 365 |

Supplementary Table 6. Ion mobility drift calculations for cluster 5 (C5).

| **Primary ID** | **Feature ID** | **m/z** | **RT** | **t_D_ (ms)** | **t_transfer_** | **t'd** | **ln t'd** | **µ (N_2_)** | **ln Ώ (N_2_)** | **Ώ (N_2_)** | **CCS (Å)** | **Ion mobility K (m/sec2)** |
| --- | --- | --- | --- | --- | --- | --- | --- | --- | --- | --- | --- | --- |
| Pos_22 | 1.40_161.0935 | 161.09 | 1.40 | 1.62 | 1.28 | 1.26 | 0.23 | 23.86 | 6.45 | 631.22 | 129.23 | 302 |
| Pos_59 | 1.92_245.1141 | 245.11 | 1.92 | 2.81 | 2.47 | 2.45 | 0.89 | 25.13 | 6.75 | 850.64 | 169.67 | 155 |
| Pos_60 | 1.92_258.1458 | 258.15 | 1.92 | 2.54 | 2.20 | 2.18 | 0.78 | 25.27 | 6.69 | 806.95 | 160.54 | 175 |
| LTNeg_9 | 1.98_258.1469 | 258.15 | 1.98 | 2.54 | 2.20 | 2.18 | 0.78 | 25.27 | 6.69 | 806.95 | 160.54 | 175 |
| Pos_71 | 2.00_233.1142 | 233.11 | 2.00 | 2.48 | 2.14 | 2.12 | 0.75 | 25.00 | 6.68 | 797.05 | 159.40 | 180 |
| Pos_77 | 2.01_269.1623 | 269.16 | 2.01 | 2.86 | 2.52 | 2.49 | 0.91 | 25.37 | 6.75 | 858.26 | 170.41 | 152 |
| Pos_99 | 2.19_189.1241 | 189.12 | 2.19 | 1.89 | 1.55 | 1.53 | 0.42 | 24.39 | 6.53 | 688.52 | 139.40 | 249 |
| Pos_130 | 3.30_189.1243 | 189.12 | 3.30 | 1.89 | 1.55 | 1.53 | 0.42 | 24.39 | 6.53 | 688.52 | 139.40 | 249 |
| Pos_146 | 3.43_247.1300 | 247.13 | 3.43 | 2.59 | 2.25 | 2.23 | 0.80 | 25.16 | 6.70 | 815.32 | 162.56 | 171 |
| Pos_185 | 3.67_215.1398 | 215.14 | 3.67 | 2.21 | 1.87 | 1.85 | 0.61 | 24.78 | 6.62 | 749.74 | 150.61 | 206 |
| Neg_63 | 3.75_259.1286 | 259.13 | 3.75 | 2.59 | 2.25 | 2.23 | 0.80 | 25.27 | 6.70 | 815.23 | 162.16 | 171 |
| LTNeg_21 | 3.76_261.1447 | 261.14 | 3.76 | 2.65 | 2.31 | 2.29 | 0.83 | 25.29 | 6.72 | 825.03 | 164.05 | 166 |
| Neg_66 | 3.79_201.1232 | 201.12 | 3.79 | 2.11 | 1.77 | 1.75 | 0.56 | 24.58 | 6.59 | 731.34 | 147.50 | 217 |
| Pos_238 | 3.93_239.1042 | 239.10 | 3.93 | 2.54 | 2.20 | 2.18 | 0.78 | 25.07 | 6.69 | 807.09 | 161.19 | 175 |
| Pos_253 | 4.03_283.1299 | 283.13 | 4.03 | 3.02 | 2.68 | 2.65 | 0.98 | 25.49 | 6.78 | 882.50 | 174.81 | 143 |
| Pos_270 | 4.08_290.1713 | 290.17 | 4.08 | 2.97 | 2.63 | 2.60 | 0.96 | 25.54 | 6.77 | 874.94 | 173.13 | 146 |
| Pos_296 | 4.19_274.2134 | 274.21 | 4.19 | 3.56 | 3.22 | 3.19 | 1.16 | 25.41 | 6.87 | 959.22 | 190.29 | 119 |
| Pos_298 | 4.20_361.2074 | 361.21 | 4.20 | 3.62 | 3.28 | 3.25 | 1.18 | 25.99 | 6.87 | 966.82 | 189.64 | 117 |
| Pos_318 | 4.32_203.1397 | 203.14 | 4.32 | 2.16 | 1.82 | 1.80 | 0.59 | 24.61 | 6.61 | 740.66 | 149.29 | 211 |
| Pos_337 | 4.40_219.1348 | 219.13 | 4.40 | 2.43 | 2.09 | 2.07 | 0.73 | 24.83 | 6.67 | 788.64 | 158.26 | 184 |
| Pos_342 | 4.44_246.1456 | 246.15 | 4.44 | 2.59 | 2.25 | 2.23 | 0.80 | 25.15 | 6.70 | 815.33 | 162.59 | 171 |
| Pos_358 | 4.55_281.1504 | 281.15 | 4.55 | 2.92 | 2.58 | 2.55 | 0.94 | 25.47 | 6.77 | 867.40 | 171.88 | 149 |
| Pos_364 | 4.59_132.1027 | 132.10 | 4.59 | 1.40 | 1.06 | 1.04 | 0.04 | 23.11 | 6.36 | 579.45 | 120.54 | 365 |
| Neg_109 | 4.61_201.1232 | 201.12 | 4.61 | 2.11 | 1.77 | 1.75 | 0.56 | 24.58 | 6.59 | 731.34 | 147.50 | 217 |
| Pos_371 | 4.61_203.1399 | 203.14 | 4.61 | 2.16 | 1.82 | 1.80 | 0.59 | 24.61 | 6.61 | 740.66 | 149.29 | 211 |
| Pos_438 | 4.99_360.2133 | 360.21 | 4.99 | 3.62 | 3.28 | 3.25 | 1.18 | 25.99 | 6.87 | 966.82 | 189.66 | 117 |
| Pos_461 | 5.10_223.1090 | 223.11 | 5.10 | 2.38 | 2.04 | 2.02 | 0.70 | 24.88 | 6.66 | 779.96 | 156.36 | 188 |
| Pos_469 | 5.14_263.1432 | 263.14 | 5.14 | 2.75 | 2.41 | 2.39 | 0.87 | 25.31 | 6.73 | 841.07 | 167.17 | 159 |
| Neg_129 | 5.17_227.1033 | 227.10 | 5.17 | 2.27 | 1.93 | 1.91 | 0.65 | 24.93 | 6.63 | 760.50 | 152.31 | 199 |
| Pos_472 | 5.18_295.1662 | 295.17 | 5.18 | 3.29 | 2.95 | 2.92 | 1.07 | 25.58 | 6.83 | 921.74 | 182.25 | 130 |
| Pos_477 | 5.20_237.1242 | 237.12 | 5.20 | 2.54 | 2.20 | 2.18 | 0.78 | 25.05 | 6.69 | 807.10 | 161.27 | 175 |
| Pos_502 | 5.33_334.1801 | 334.18 | 5.33 | 3.29 | 2.95 | 2.92 | 1.07 | 25.84 | 6.83 | 921.52 | 181.28 | 130 |
| Pos_506 | 5.35_231.1713 | 231.17 | 5.35 | 2.00 | 1.66 | 1.64 | 0.49 | 24.98 | 6.57 | 709.98 | 142.05 | 232 |
| Pos_508 | 5.37_263.1433 | 263.14 | 5.37 | 2.75 | 2.41 | 2.39 | 0.87 | 25.31 | 6.73 | 841.07 | 167.17 | 159 |
| Pos_523 | 5.44_275.1609 | 275.16 | 5.44 | 2.86 | 2.52 | 2.49 | 0.91 | 25.42 | 6.75 | 858.22 | 170.22 | 152 |
| Neg_142 | 5.52_356.1437 | 356.14 | 5.52 | 3.67 | 3.33 | 3.30 | 1.19 | 25.96 | 6.88 | 973.50 | 191.05 | 115 |
| Pos_538 | 5.58_263.1407 | 263.14 | 5.58 | 2.75 | 2.41 | 2.39 | 0.87 | 25.31 | 6.73 | 841.07 | 167.17 | 159 |
| Pos_581 | 5.85_263.1432 | 263.14 | 5.85 | 2.75 | 2.41 | 2.39 | 0.87 | 25.31 | 6.73 | 841.07 | 167.17 | 159 |
| Pos_584 | 5.89_275.1612 | 275.16 | 5.89 | 2.86 | 2.52 | 2.49 | 0.91 | 25.42 | 6.75 | 858.22 | 170.22 | 152 |
| Pos_599 | 5.99_265.1558 | 265.16 | 5.99 | 2.81 | 2.47 | 2.44 | 0.89 | 25.33 | 6.75 | 850.50 | 168.99 | 155 |
| Neg_170 | 6.32_241.1183 | 241.12 | 6.32 | 2.32 | 1.98 | 1.96 | 0.67 | 25.09 | 6.65 | 769.29 | 153.58 | 194 |
| Pos_644 | 6.35_243.1563 | 243.16 | 6.35 | 2.38 | 2.04 | 2.02 | 0.70 | 25.11 | 6.66 | 779.80 | 155.61 | 189 |
| Pos_645 | 6.36_245.1870 | 245.19 | 6.36 | 2.81 | 2.47 | 2.45 | 0.89 | 25.14 | 6.75 | 850.64 | 169.67 | 155 |
| Neg_171 | 6.38_243.1703 | 243.17 | 6.38 | 2.70 | 2.36 | 2.34 | 0.85 | 25.11 | 6.73 | 833.23 | 166.27 | 163 |
| Pos_649 | 6.41_231.1715 | 231.17 | 6.41 | 2.00 | 1.66 | 1.64 | 0.49 | 24.98 | 6.57 | 709.98 | 142.05 | 232 |
| Neg_181 | 6.60_241.1182 | 241.12 | 6.60 | 2.32 | 1.98 | 1.96 | 0.67 | 25.09 | 6.65 | 769.29 | 153.58 | 194 |
| Pos_688 | 6.75_279.1714 | 279.17 | 6.75 | 2.75 | 2.41 | 2.38 | 0.87 | 25.45 | 6.73 | 840.96 | 166.69 | 159 |
| Pos_727 | 7.08_309.1820 | 309.18 | 7.08 | 2.48 | 2.14 | 2.11 | 0.75 | 25.68 | 6.68 | 796.50 | 157.18 | 180 |
| Pos_735 | 7.18_275.1610 | 275.16 | 7.18 | 2.86 | 2.52 | 2.49 | 0.91 | 25.42 | 6.75 | 858.22 | 170.22 | 152 |
| Pos_742 | 7.25_231.1713 | 231.17 | 7.25 | 2.59 | 2.25 | 2.23 | 0.80 | 24.98 | 6.70 | 815.44 | 163.15 | 171 |
| Pos_743 | 7.26_257.1868 | 257.19 | 7.26 | 2.70 | 2.36 | 2.34 | 0.85 | 25.26 | 6.73 | 833.13 | 165.78 | 163 |
| Pos_790 | 8.13_291.1725 | 291.17 | 8.13 | 2.81 | 2.47 | 2.44 | 0.89 | 25.55 | 6.75 | 850.33 | 168.23 | 155 |
| Pos_794 | 8.18_259.2028 | 259.20 | 8.18 | 2.97 | 2.63 | 2.61 | 0.96 | 25.28 | 6.77 | 875.14 | 174.07 | 146 |
| Pos_799 | 8.34_323.1971 | 323.20 | 8.34 | 3.67 | 3.33 | 3.30 | 1.19 | 25.77 | 6.88 | 973.67 | 191.79 | 115 |
| Pos_809 | 8.95_273.2181 | 273.22 | 8.95 | 3.08 | 2.74 | 2.71 | 1.00 | 25.40 | 6.79 | 891.48 | 176.88 | 140 |
| Pos_812 | 9.11_273.2182 | 273.22 | 9.11 | 3.08 | 2.74 | 2.71 | 1.00 | 25.40 | 6.79 | 891.48 | 176.88 | 140 |
| Pos_819 | 9.64_307.2022 | 307.20 | 9.64 | 3.46 | 3.12 | 3.09 | 1.13 | 25.67 | 6.85 | 945.41 | 186.61 | 123 |
